# A generalized Gompertz growth model with applications and related birth-death processes

**DOI:** 10.1007/s11587-020-00548-y

**Published:** 2020-12-23

**Authors:** Majid Asadi, Antonio Di Crescenzo, Farkhondeh A. Sajadi, Serena Spina

**Affiliations:** 1 Department of Statistics, Faculty of Mathematics and Statistics, University of Isfahan, Isfahan, 81744 Iran; 2 School of Mathematics, Institute for Research in Fundamental Sciences (IPM), P.O. Box 19395, Tehran, Iran; 3 Dipartimento di Matematica, Università degli Studi di Salerno, Via Giovanni Paolo II, 132, 84084 Fisciano, SA Italy

**Keywords:** Gompertz model, Birth-death process, Ultimate extinction probability, First-passage-time problem

## Abstract

In this paper, we propose a flexible growth model that constitutes a suitable generalization of the well-known Gompertz model. We perform an analysis of various features of interest, including a sensitivity analysis of the initial value and the three parameters of the model. We show that the considered model provides a good fit to some real datasets concerning the growth of the number of individuals infected during the COVID-19 outbreak, and software failure data. The goodness of fit is established on the ground of the ISRP metric and the $d_2$-distance. We also analyze two time-inhomogeneous stochastic processes, namely a birth-death process and a birth process, whose means are equal to the proposed growth curve. In the first case we obtain the probability of ultimate extinction, being 0 an absorbing endpoint. We also deal with a threshold crossing problem both for the proposed growth curve and the corresponding birth process. A simulation procedure for the latter process is also exploited.

## Introduction

Constructing growth curves describing dynamic evolutions is relevant to several applied fields. Indeed, growth dynamics are exhibited by a large number of real systems, such as the number of individual infected during an epidemic, the size of a living body during juvenility, other indicators associated with population growth, etc. The basic reference in this respect is the well-known Malthusian model, governed by the differential equation

1 $$\begin{aligned} \frac{d \, N_M(t)}{dt}= r N_M(t), \quad t>0, \end{aligned}$$

where $N_M(t)$ represents the population size and $r > 0$ is the growth rate. This leads to exponential curve, adopted often in population ecology to describe growth in absence of constraints. Mathematical models associated to phenomena governed by growth tendency are based on suitable refinements of the exponential curve, such as the logistic and its generalizations treated in Tsoularis and Wallace [35]. Indeed, the traditional models are not always appropriate to describe certain growth phenomena since they involve few parameters. On the contrary, models involving more parameters are much more flexible and provide in general a better fit of the observed data. In this framework it is worth mentioning the contribution by Wu et al. [37], that adopted a model based on the generalized Richards curve for the COVID-19 outbreak. The latter curve is governed by the differential equation

$$\begin{aligned} \frac{d \, N_R(t)}{dt}= r[N_R(t)]^p \left[ 1-\left( \frac{N_R(t)}{C}\right) ^{\alpha }\right] , \quad t>0, \end{aligned}$$

where $N_R(t)$ represents the cumulative number of cases at time *t*, *r* is the growth rate at the early stage, *C* is the carrying capacity (i.e., the final epidemic size), $p\in [0,1]$ and $\alpha$ are suitable shape parameters. Other forms of generalized growth models can be found in further investigations. For instance, Rincón et al. [29] analyzed a generalized Fujikawa’s growth model described by the equation

$$\begin{aligned} \frac{d \, N_F(t)}{dt}= r[N_F(t)]^\alpha \left[ 1-\left( \frac{N_F(t)}{C}\right) ^{\gamma }\right] \left[ 1-\left( \frac{N_m}{N_F(t)}\right) ^{c}\right] , \quad t>0, \end{aligned}$$

where *r*, $\alpha$, $\gamma$, *c*, *C* and $N_m$ are constants, with $r>0$, $K>N_m>0$, and *C* is the carrying capacity. Another case of interest has been treated recently by Chakraborty et al. [9] to propose a generalization of simple equations modeling the growth mechanism of biological processes, and finalized to generate more flexible shapes. Furthermore, a detailed treatment of extensions of the Gompertz-type equation in modern science has been presented in the book by Kyurkchiev and Iliev [24].

Along the lines of the above mentioned researches, the present paper is aimed to propose a suitable extension of the celebrated Gompertz model. This is a well-known growth model that is frequently adopted among the sigmoid models for fitting real data, and is governed by the following differential equation:

2 $$\begin{aligned} \frac{d \, N_G(t)}{dt}=N_G(t) \left( \alpha - \beta \log \frac{N_G(t)}{y}\right) , \quad t>0, \qquad N_G(0)=y>0, \end{aligned}$$

with $\alpha ,\beta >0$. Various re-parametrisations of this model have been considered by Tjørve and Tjørve [34]. The generalization proposed herewith is based on a suitable exponentiation of the term in parenthesis on the right-hand-side of (2). This leads to a more flexible growth model, which includes a variety of cases for the asymptotic analysis. Indeed, it may tend to infinity, or to a finite limit (the carrying capacity), or to zero. The resulting model provides also a generalization of a modified Korf model investigated recently in Di Crescenzo and Spina [12]. For a complete analysis of the model we study various features of interest, such as the correction factor, the relative growth rate, the inflection point, the maximum specific growth rate and the lag time. For the proposed model we also face a threshold crossing problem and perform a sensitivity analysis of the parameters.

We purpose to analyze also a stochastic counterpart of the proposed model. This is motivated by the need of describing growth phenomena subject to random perturbations by means of mathematical models based on stochastic processes. Specifically, a desirable characteristics is that the mean evolution of the process coincides with the deterministic curve of the proposed model. In this framework, stochastic processes for the modeling of real growth phenomena have been largely considered in the literature. We recall the well-known approach based on diffusion processes for the stochastic model of tumor growth, such as that exploited in Albano and Giorno [1], Giorno et al. [18], Giorno and Nobile [16], Hanson and Tier [20], Spina et al. [31]. Other studies including Gompertz and logistic growth models based on stochastic diffusions can be found in Campillo et al. [8], Himadri Ghosh and Prajneshu [22], and Yoshioka et al. [38]. Recent advances involving fractional Gompertz growth models in biological contexts have been analyzed in Ascione and Pirozzi [4], Dewanji et al. [11], Frunzo et al. [15], and in Meoli et al. [25]. Nevertheless, our analysis will be restricted to the case of birth-death processes. The usefulness of this family of stochastic processes in the applications to sciences has been described recently in Crawford and Suchard [10]. We recall some previous investigations oriented to the analysis of birth-death processes for logistic and Gompertz stochastic growth, such as Di Crescenzo and Paraggio [13], Parthasarathy and Krishna Kumar [27], Swift [32] and Tan [33]. Various computational issues and a first-passage-time problem for time-inhomogeneous birth-death processes are investigated in Giorno and Nobile [17].

First we study an inhomogeneous birth-death process with linear time-dependent birth and death rates. The analysis is also developed towards the more interesting case of a time-inhomogeneous linear birth process. In both cases we specify the conditions that allow the mean of the process to be identical to the proposed generalized Gompertz growth curve.

In order to pinpoint the usefulness of the proposed model and the given results we focus on some applications to real data. Indeed, we show that the considered growth model is able to provide a good fit to various datasets in real cases of interest. As a first application we consider the data of the active and of the total number of contagions over a given period of the COVID-19 outbreak spread in Iran and Italy. Specifically, we analyze the proposed model, the Gompertz model and the logistic model as candidates to fit the considered data. We show that, among the cases under investigation, in many instances the proposed model provides the better fit under two comparison criteria. Indeed, in order to assess the goodness of the curve fitting, we adopt the ISRP metric (introduced recently in [5]) and the $d_2$-distance. We point out that this investigation is finalized to perform a detailed investigation on an extended Gompertz growth model, rather than to solve the complex problem of finding a proper mathematical model to describe the evolution of the COVID-19 outbreak. The second application is devoted to software failure data from Tandem Computers. Also in this case, it is shown that the proposed model provides the better fit of the considered data under the ISRP metric and the $d_2$-distance. This confirms that the model is effective for various instances of growth dynamics.

Let us now describe the plan of the paper. In Sect. 2 we introduce the model and illustrate its mean features, including the different shapes exhibited by the growth curve according to the parameters. Section 3 is devoted to a detailed analysis of the relevant features of the model. In Sect. 4 we then provide the announced applications of the proposed model to the number of outbreak contagions and to software failure data. In Sect. 5 we analyze a special inhomogeneous linear birth-death process, and provide a condition on the time dependent terms of the birth and death rates such that the mean of the process is equal to the proposed growth curve. A special case is treated in Sect. 6, where the case of a pure time-inhomogeneous birth process is considered. We also face the first-passage-time problem of this process through constant boundaries and time-varying boundaries. The latter case is developed by means of a simulation-based approach. To this aim a procedure able to simulate the birth times of the process is sketched. Some concluding remarks are finally provided in Sect. 7.

## The proposed model

In this paper, we propose and study the growth model $N_{GPD}(t)$, which is solution of the following differential equation:

3 $$\begin{aligned} \frac{d\,N_{GPD}(t)}{dt} =N_{GPD}(t) \,A \left( 1-\frac{1}{A b} \log \frac{N_{GPD}(t)}{y}\right) ^{ 1+ a}, \quad t>0,\quad N_{GPD}(0)=y, \end{aligned}$$

with $y>0$, $A>0$, $a >-1$, $a\ne 0$ and $b > 0$. This is a suitable extension of the Gompertz model governed by Eq. (2). Moreover, such a model is included in the family of growth models $N(t),\,t>0$, that are described by a different kind of differential equation, namely

4 $$\begin{aligned} \frac{d\,N(t)}{dt}=\xi (t) N(t),\qquad t>0, \end{aligned}$$

where *N*(*t*) denotes the size of a population at time *t* and $\xi (t)>0$ is a time-dependent growth rate function. Suitable choices of the growth rate $\xi (t)$ allow us to define a variety of models of interest. For instance,

- the constant rate $\xi (t) = r$ yields the classical Malthusian growth (cf. Eq. (1)):
  $$\begin{aligned} N_M(t) = y e^{r t}, \quad t> 0, \qquad N_M(0)=y>0; \end{aligned}$$
- the rate $\xi (t) = \xi _G(t):=\alpha e^{-\beta t}$ refers to the Gompertz growth model (cf. Gompertz [19])
  5 $$\begin{aligned} N_G(t) = y \exp \left\{ \frac{\alpha }{\beta }(1-e^{-\beta t})\right\} , \quad t> 0, \qquad N_G(0)=y>0,\;\alpha ,\beta >0; \end{aligned}$$
- the rate $\xi (t) =\xi _K(t):= \alpha t^{-(\beta +1)}$ is concerning the Korf growth model (cf. Korf [23])
  6 $$\begin{aligned} N_K(t) = y \exp \left\{ \frac{\alpha }{\beta }(1-t^{-\beta })\right\} , \quad t> 0, \qquad N_K(0)=0,\;\alpha ,\beta >0; \end{aligned}$$
- the rate $\xi (t) = \xi _{DS}(t):= \alpha (1+t)^{-(\beta +1)}\equiv \xi _K(1+t)$, with $\alpha , \beta >0$, leads to a recently proposed growth model (cf. Di Crescenzo and Spina [12])
  7 $$\begin{aligned} N_{DS}(t)=y \exp \left\{ \frac{\alpha }{\beta } \left[ 1-\left( 1+t\right) ^{-\beta }\right] \right\} , \quad t> 0, \qquad N_{DS}(0) = y > 0. \end{aligned}$$

Let us now consider a suitable family of growth models, whose growth rate function is defined as follows:

8 $$\begin{aligned} \xi (t)=A \,\bar{F}(t), \qquad t>0, \end{aligned}$$

where $\bar{F}(t)={\mathbb {P}}(X>t)$ is the survival function of a given nonnegative continuous random variable *X*, and $A>0$ is a constant. From the differential equation (4) and Eq. (8), we have that the corresponding growth curve is given by

9 $$\begin{aligned} N(t)=y \exp \left\{ A \int _0^{t} \bar{F}(\tau )\,\mathrm{d}\tau \right\} , \qquad t>0, \qquad N(0)=y>0. \end{aligned}$$

Hereafter we show that the representation (9) allows to express some properties of *N*(*t*) in terms of the distribution of *X*.

### Remark 1

The ultimate behaviour of the model (9) depends on the nature of the expectation of *X*, denoted as $m:={\mathbb {E}}[X]=\int _0^{\infty } \bar{F}(\tau )\,\mathrm{d}\tau$. Indeed, one has

$$\begin{aligned} N(t) \quad {\mathop {\rightarrow }\limits ^{t\rightarrow +\infty }} \quad \left\{ \begin{array}{ll} +\infty , &{} \hbox { if }m=+\infty , \\ y e^{A m}=: C, &{} \hbox { if }m<+\infty , \end{array}\right. \end{aligned}$$

where *C* is finite and denotes the carrying capacity of *N*(*t*).

Let us now recall the notion of increasing concave ordering (see Section 4.A of Shaked and Shanthikumar [30]). Given two random variables $X_i$, $i=1,2$, having distribution functions $F_i(t)=1-\bar{F}_i(t)$, $i=1,2$, we say that $X_1$ is smaller than $X_2$ in the increasing concave order (denoted by $X_1 \le _\mathrm{icv} X_2$) if $\int _{-\infty }^t F_1(\tau )\,\mathrm{d}\tau \ge \int _{-\infty }^t F_2(\tau )\,\mathrm{d}\tau$ for all $t\in {\mathbb {R}}$. Roughly speaking, this means that $X_1$ is both “smaller” and “more variable” than $X_2$ in some stochastic sense.

### Remark 2

The increasing concave order allows to compare growth models of the form (9). For $i=1,2$, let

$$\begin{aligned} N_i(t)=y_i \exp \left\{ A_i \int _0^{t} \bar{F}_i(\tau )\,\mathrm{d}\tau \right\} , \qquad t>0, \qquad N_i(0)=y_i>0. \end{aligned}$$

It is not hard to see that if $y_1 \le y_2$, $A_1 \le A_2$, and $X_1 \le _\mathrm{icv} X_2$, then $N_1(t)\le N_2(t)$ for all $t\ge 0$.

In order to propose a flexible growth model representing a generalization of various previous instances, we assume that *X* has a generalized Pareto distribution (GPD) with survival function

10 $$\begin{aligned} \bar{F}(t)=\left( \frac{b}{at+b}\right) ^{\frac{1}{a}+1},\qquad t\ge 0, \end{aligned}$$

where $a>-1$, $a\ne 0$, $b>0$. We recall that some characterization results on the GPD can be found in Asadi et al. [3], in Section 4 of Hashemi et al. [21] and in Section 3.1 of Arriaza et al. [2]. In particular, this family includes three distributions, depending on the values of *a*:

- the Pareto distribution, when $a>0$;
- the Power distribution, when $-1<a<0$ (in this case the distribution is bounded above); in particular, the distribution is Uniform if $a=\frac{1}{2}$;
- the Exponential distribution, when $a\rightarrow 0$.

Let $A>0$, $a>-1$, $a\ne 0$, $b>0$. Due to Eqs. (8) and (9), the model driven by the GPD survival function (10) has respectively growth rate function

11 $$\begin{aligned} \xi (t)=A \left( \frac{b}{at+b}\right) ^{\frac{1}{a}+1}, \qquad t>0, \end{aligned}$$

and growth curve

12 $$\begin{aligned} N_{GPD}(t)=y\,\exp \left\{ Ab\left[ 1-\left( \frac{b}{at+b} \right) ^{\frac{1}{a}}\right] \right\} , \qquad t>0, \end{aligned}$$

with initial value $N_{GPD}(0)=y>0$. Moreover, the function (12) is the solution of (3).

By taking $A=\alpha >0$, $a=b=1/\beta$ in (12), one obtains the growth model $N_{DS}(t)$ given in (7). Moreover, if $A=\alpha >0$, $b=1/\beta$ and *a* tends to 0, then the proposed model (12) gives the Gompertz curve (5).

We point out that the curve (12) exhibits different behavior according to the value of $a>-1$, $a \ne 0$. In particular, by studying the derivative

13 $$\begin{aligned} \frac{d \,N_{GPD}(t)}{dt}=A\,N_{GPD}(t)\left( \frac{b}{at+b}\right) ^{\frac{1}{a}+1}, \qquad t>0 \end{aligned}$$

the following cases arise:

(i) If $a>0$, the curve (12) is well defined for all $t\in (0,+\infty )$; it is an increasing function for all $t>0$; for $t\rightarrow +\infty$ it tends to the carrying capacity
  14 $$\begin{aligned} C=y\,e^{Ab}. \end{aligned}$$
(ii) If $-1<a<0$ and $\frac{1}{\left| a\right| }$ is an odd integer, $N_{GPD}(t)$ defined in (12) is well defined for all $t\in (0,+\infty )$, with $N_{GPD}(\frac{b}{|a|})=C=y\,e^{Ab}$; moreover, the curve $N_{GPD}(t)$ is increasing for all $t>0$, and it goes to infinity for $t\rightarrow +\infty$.
(iii) When $-1<a<0$ and $\frac{1}{\left| a\right| }$ is an even integer, $N_{GPD}(t)$ is well defined for all $t\in (0,+\infty )$. Specifically, $N_{GPD}(t)$ is increasing in $0<t\le \frac{b}{|a|}$, with maximum $N_{GPD}(\frac{b}{|a|})=C=y\,e^{Ab}$, then it is decreasing for $t\ge \frac{b}{|a|}$ and tends to zero for $t\rightarrow +\infty$.
(iv) If $-1<a<0$ and $\frac{1}{a}$ is a non-integer real number, $N_{GPD}(t)$ is defined only for $t\in (0,\frac{b}{|a|})$, with $\lim _{t\rightarrow \left( \frac{b}{|a|}\right) ^-} N_{GPD}(t)=C=ye^{Ab}$. In this case $N_{GPD}(t)$ is an increasing function for $0<t<\frac{b}{|a|}$.

**Fig. 1:**
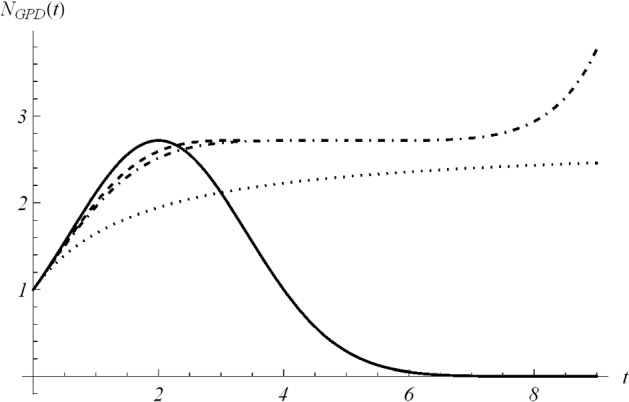
The curve $N_{GPD}(t)$, given in (12), is plotted for $y =1$, $A=1$, $b=1$ and $a=-0.5$ (solid), $a=-0.3$ (dashed), $a=-0.2$ (dot-dashed), $a=1$ (dotted)

In Fig. 1, the curve (12) is plotted for different choices of *a*, with case (i) for $a=1$, case (ii) for $a=-0.2$, case (iii) for $a=-0.5$, and case (iv) for $a=-0.3$. Note that when $a=-0.3$, for case (iv), the curve is defined only in $0<t<3.\bar{3}=b/|a|$ as specified above. Moreover, if $a=-0.5$, the random variable *X* considered in (8) is uniformly distributed; in this case the population size $N_{GPD}(t)$ tends to zero, i.e. to the extinction.

### Remark 3

From (10) it is not hard to see that for the GPD distribution one has that $\int _{0}^t F(\tau )\,\mathrm{d}\tau$ is increasing in $a\in (-1,0)\cup (0,+\infty )$ and is decreasing in $b\in (0,+\infty )$ for all $t\ge 0$. Hence, denoting by $N_{GPD, i}(t)$ the growth model (12) characterized by parameters $y_i, A_i,a_i,b_i$, $i=1,2$, due to Remark 2 we have that if $y_1 \le y_2$, $A_1 \le A_2$, $a_1 \ge a_2$ and $b_1 \le b_2$, then

$$\begin{aligned} N_{GPD, 1}(t)\le N_{GPD, 2}(t)\quad \hbox {for all}\ t\ge 0. \end{aligned}$$

## Analysis of the growth model

In this section we discuss various features of the generalized Gompertz growth model proposed in (12).

### The correction factor and the relative growth rate

In the context of growth analysis, interest is given to models governed by equations of the following form:

$$\begin{aligned} \frac{d\,N(t)}{dt}=A\, N (t) f\left[ N(t)\right] , \qquad t>0. \end{aligned}$$

The function *f* is a function of *t* only through the population size *N*(*t*), and is named a size covariate model. It allows to express the density dependent growth of the curve, and it is strictly related to the *relative growth rate* *g* (see Tsoularis and Wallace [35]) through the following relation:

$$\begin{aligned} g[N(t)]:=\frac{1}{N(t)}\frac{d\,N(t)}{dt}\equiv A\, f[N(t)], \qquad t>0. \end{aligned}$$

We remark that an extension of the relative growth rate, named modified relative growth rate, has been proposed and studied recently in Pal et al. [26] for the data analysis of growth models. Moreover, the function *f* is also called *correction factor* because it provides information about the deviation of a growth model from the classical exponential growth (for which $f(z)=1$, $z\ge 0$). From Eqs. (3) one has that for the GPD model the correction factor is given by

$$\begin{aligned} f(z)= \left( 1-\frac{1}{Ab}\log \frac{z}{y}\right) ^{ 1+a}, \qquad z>0. \end{aligned}$$

**Fig. 2:**
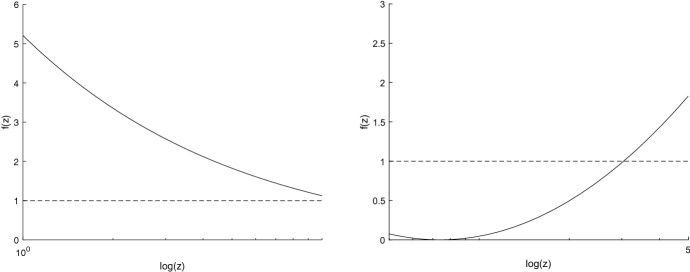
The correction factor for model (12) (solid line), for $y = 0.1$, $A = 3,a=b=10$ on the left and $A =2,a=b=1$ on the right, compared with $f(z)=1$ (dashed line) for the exponential growth

As example, Fig. 2 shows some plots of the correction factor for model (12).

### The inflection point

The monotonicity of the proposed model (12) has been studied in Sect. 2. Now we focus on the second derivative of $N_{GPD}(t)$ in order to compute the inflection point, when existing. From (13), the second derivative of $N_{GPD}(t)$, $t>0$, results:

15 $$\begin{aligned} \frac{d^2 N_{GPD}(t)}{dt^2} = {A}\,N_{GPD}(t)\left( \frac{b}{at+b}\right) ^{\frac{1}{a}+1} \left[ A \left( \frac{b}{at+b}\right) ^{\frac{1}{a}+1}-\frac{a+1}{at +b}\right] . \end{aligned}$$

For the study of the sign of (15), by considering the analysis shown in Sect. 2 we report two cases:

- If $Ab>a+1$, in cases (i), (iii) and (iv), the curve $N_{GPD}(t)$ is sigmoidal with the inflection point given by
  16 $$\begin{aligned} t_I:=\frac{b}{a}\left[ \left( \frac{Ab}{a+1}\right) ^{a}-1\right] . \end{aligned}$$
  On the contrary, in case (ii) the curve $N_{GPD}(t)$ has two inflection points, i.e. $t_I$ and $\frac{b}{|a|}$, with $t_I<\frac{b}{|a|}$. In particular, it has an upward concavity up to $t=t_I$, a downward concavity between $t_I$ and $\frac{b}{|a|}$, then an upward concavity.
- If $Ab<a+1$, in cases (i), (iii) and (iv), the curve $N_{GPD}(t)$ has a downward concavity for all $t>0$, whereas in case (ii), $N_{GPD}(t)$ has a downward concavity up to the inflection point $\frac{b}{|a|}$, then it has an upward concavity.

Furthermore, in all the interesting cases, the population at time $t=t_I$ is given by

$$\begin{aligned} N_{GPD}\left( t_I\right) =C\, e^{-(a+1)}, \end{aligned}$$

where *C* is given in (14).

### The maximum specific growth rate and the lag time

The study of a growth curve that exhibits a sigmoidal behaviour in proximity of its inflection point (by approximating the curve in that point with a line) can be of interest in many phenomena that show lag, growth, and asymptotic phases. Due to the results of Sect. 3.2, we focus only on the instances of interest, that are cases (i), (iii) and (iv) analysed in Sect. 2, when $Ab>a+1$.

We consider the *maximum specific growth rate*, say $\mu$, which is the coefficient of the tangent to the curve $N_{GPD}(t)$ given in (12) in the inflection point $t_I$ shown in (16):

17 $$\begin{aligned} \mu :=\frac{d\,N_{GPD}(t)}{dt}\Big |_{t=t_I} =A y\left( \frac{a+1}{Ab}\right) ^{ 1+a}e^{Ab-(a+1)}. \end{aligned}$$

Moreover, recalling the expressions of $t_I$ and $N_{GPD}(t_I)$, the tangent to the curve $N_{GPD}(t)$ in $\left( t_I,\,N_{GPD}(t_I) \right)$ is

18 $$\begin{aligned} n:=\mu t+y e^{Ab-a-1}\left[ \left( \frac{a+1}{Ab}\right) ^{a} \frac{a+1}{a}-\frac{1}{a}\right] , \end{aligned}$$

with $\mu$ expressed in (17). (For notation clarity, we denote by *n* the *y*-axis). Moreover, we introduce the lag time $\lambda$ as the *x*-axis intercept of the tangent line. The lag time $\lambda$ for the model (12) is the *t*-axis intercept of (18), that is

19 $$\begin{aligned} \lambda =\frac{b}{a}\left[ \left( \frac{Ab}{a+1}\right) ^{ a} \frac{1}{a+1}-1\right] . \end{aligned}$$

**Fig. 3:**
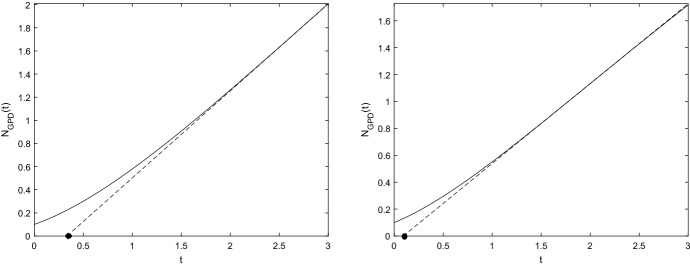
The tangent lines (dotted curves) and the lag times (black point) with $y=0.1$, $A=3,\,a=b=2$ on the left and $A=3,\,a=b=1.7$ on the right

Note that $\lambda$ is positive when $Ab>(a+1)^{\frac{1}{a}+1}$. Hence, the sigmoidal function $N_{GPD}(t)$ evolves with a growth rate that starts at zero and then accelerates to the maximal value $\mu$, given in (17), in the time period resulting in the lag time $\lambda$ shown in (19). Examples of tangent lines (dotted curves) and lag times (black point) are plotted in Fig. 3.

### Threshold crossing

In several contexts of population evolution, it is relevant to know how long time the population spends below (or above) a certain preassigned value, which can represent a control threshold or a boundary in the evolution of the given phenomenon. Let us denote by *S* such a threshold for a growth model *N*(*t*). We are interested in the time instant in which *N*(*t*) reaches *S*, with $S>N(0)=y$. Taking into account the various behaviours that the curve $N_{GPD}(t)$ may have for different choices of *a*, we are interested in a generic threshold $S>y$ if the curve grows to infinity, or on a percentage *p* of the carrying capacity $C=ye^{Ab}$, or on a percentage of the size of the population at the inflection point $t_I$ given in (16). For a generic *S*, to obtain the crossing time instant $\theta ^S$ of $N_{GPD}(t)$ through *S*, we solve the equation $N(\theta ^S)=S$, recalling (12). Thus we get the solution

20 $$\begin{aligned} \theta ^S=\frac{b}{a}\left[ \left( 1-\frac{1}{Ab} \log \frac{S}{y}\right) ^{-a}-1\right] , \qquad S>y. \end{aligned}$$

If *S* is a percentage $0<p<1$ of the carrying capacity $C=y\,e^{Ab}$, substituting $S=pC$ in (20), the time of interest is

$$\begin{aligned} \theta _C(p)=\frac{b}{a}\left[ \left( -\frac{1}{Ab} \log p\right) ^{-a}-1\right] , \qquad e^{-Ab}<p<1. \end{aligned}$$

Moreover, when *S* is a percentage $0<p<1$ of $N_{GPD}(t_I)$, i.e. $S=p\,y\,e^{Ab-a-1}$, one has that the crossing time is:

$$\begin{aligned} \theta _I(p)=\frac{b}{a}\left[ \left( -\frac{1}{Ab} \left( \log p-a-1\right) \right) ^{-a}-1\right] , \qquad e^{-Ab+a+1}<p<1, \;Ab>a+1. \end{aligned}$$

**Fig. 4:**
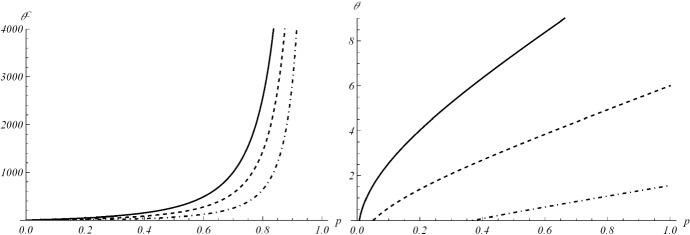
The threshold crossing times $\theta _C(p)$ (on the left) and $\theta _I(p)$ (on the right) are plotted as function of *p*, with $y=0.1$, $a=2$, $b=4$ and $A=2$, 1.5, 1, from top to bottom

In Fig. 4, the quantities $\theta _C(p)$ and $\theta _I(p)$ are plotted as a function of *p*, with some choices of the parameters.

### Sensitivity analysis

This section is devoted to analyze how the perturbation on each parameter involved in the model (12) influences the growth of $N_{GPD}(t)$. The parameters are the initial state $y>0$, then $A>0$, $a>-1$, $a \ne 0$, and $b>0$. Recalling the study performed in Sect. 2 about the conditions for the existence, the following analysis can be performed on $N_{GPD}(t)$, that hereafter will be denoted by $N_{GPD}^\nu$ to emphasize the dependence on a generic parameter $\nu$. Specifically, starting from (12) we expand $N_{GPD}^{\nu +\epsilon }$ in a Taylor series evaluated at $\nu$, with $\epsilon >0$, for $\nu =y$, *A*, *a* and *b*.

- *Perturbation on* *y*
  $$\begin{aligned} N_{GPD}^{y+\epsilon }-N_{GPD}^{y}\approx \epsilon \, \exp \left\{ Ab\left[ 1-\left( \frac{b}{at+b}\right) ^\frac{1}{a}\right] \right\} . \end{aligned}$$
  The latter term is positive for all $t>0$.
- *Perturbation on* *A*
  $$\begin{aligned} N_{GPD}^{A+\epsilon }-N_{GPD}^{A} \approx \epsilon \,N_{GPD}^{A}\,b \, \left[ 1-\left( \frac{b}{at+b}\right) ^{\frac{1}{a}}\right] . \end{aligned}$$
  The latter term is positive for all $t>0$.
- *Perturbation on* *a*
  21 $$\begin{aligned} N_{GPD}^{a+\epsilon }-N_{GPD}^{a} \approx \epsilon \,N_{GPD}^{a} \,\frac{A}{a^2} \left( \frac{b}{at+b}\right) ^{\frac{1}{a}+1} \left[ at+(at+b)\log \left( \frac{b}{at+b}\right) \right] . \end{aligned}$$
  To study the sign of (21), we note that due to the analysis performed in Sect. 2, it is not hard to see that
  $$\begin{aligned} \left( \frac{b}{at+b}\right) ^{\frac{1}{a}+1}> 0, \qquad at+(at+b)\log \left( \frac{b}{at+b}\right) < 0, \end{aligned}$$
  where the time domain is $t>0$ if $a>0$, and $0<t<\frac{b}{|a|}$ if $-1<a<0$. Hence, the right-hand-side of (21) is negative for all $t>0$.
- *Perturbation on* *b*
  22 $$\begin{aligned} N_{GPD}^{b+\epsilon }-N_{GPD}^{b}\approx \epsilon \,N_{GPD}^{b}\,A\, \left[ 1-\left( \frac{b}{at+b}\right) ^{\frac{1}{a}}\left( 1+\frac{t}{at+b}\right) \right] . \end{aligned}$$
  The sign of the right-hand-side of (22) is equal to that of the function
  $$\begin{aligned} h(t):=1-\left( \frac{b}{at+b}\right) ^{\frac{1}{a}}\left( 1+\frac{t}{at+b}\right) . \end{aligned}$$
  We have $h(0)=0$. The first derivative $h'(t)$ is positive for all $t>0$ in cases (i), (iii) and (iv) analyzed in Sect. 2; hence, in these cases one has $h(t)>0$ for all $t>0$. In the remaining case (ii), we have $h'(t)>0$ for $0<t<\frac{b}{|a|}$, and $h'(t)<0$ for $t>\frac{b}{|a|}$. Hence, the function *h*(*t*) is surely positive up to $t=\frac{b}{|a|}$. Noting that $\lim _{t\rightarrow +\infty }h(t)=-\infty$, from the continuity of *h* we have that there exists a $\bar{t}>\frac{b}{|a|}$ such that $h(\bar{t})<0$ for all $t>\bar{t}$.

**Fig. 5:**
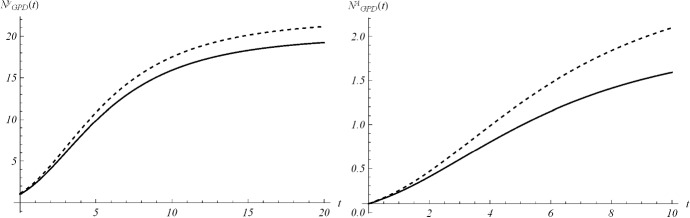
On the left, the curve $N_{GPD}(t)$ for initial value $y=1$ (solid) and $y=1.1$ (dashed), with $A=1$, $a=0.2$, $b=3$. On the right, $N_{GPD}(t)$ is plotted for $A=1$ (solid) and $A=1.1$ (dashed), with $y=0.1$, $a=0.2$, $b=3$

**Fig. 6:**
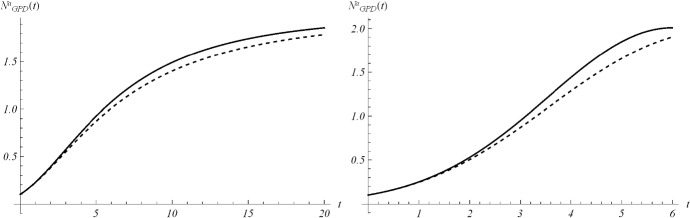
On the left, the curve $N_{GPD}(t)$ for $a=0.3$ (solid) and $a=0.4$ (dashed), with $y=0.1$, $A=1$, $b=3$. On the right, the same curve is plotted for $a=-0.5$ (solid) and $a=-0.4$ (dashed), with $y=0.1$, $A=1$, $b=3$

**Fig. 7:**
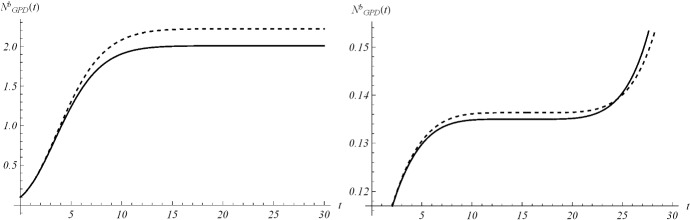
On the left, $N_{GPD}(t)$ for $b=3$ (solid) and $b+\epsilon =3.1$ (dashed), with $y=0.1$, $A=1$, $a=-0.1$. On the right, $N_{GPD}(t)$ is plotted for $b=3$ (solid) and $b+\epsilon =3.1$ (dashed), with $y=0.1$, $A=1$, $a=-0.2$

As example, in Fig. 5 we show the curve $$N_{GPD}(t)$$ and the effect of the perturbation $$\epsilon =0.1$$ on the parameter *y* (on the left) and on *A* (on the right). The same is show in Fig. 6 for the parameter *a*, when $$ a>0$$ (on the left) and $$-1<a<0$$ (on the right), and finally in Fig. 7 for the parameter *b* in case (iii) (on the left) and in case (ii) (on the right).

## Applications to real data

The usefulness of the results presented in the previous part of the paper emerges in various contexts in which the proposed generalization of the Gompertz model may be employed to obtain proper fit of real data. Indeed, in this section we show that the proposed GPD model (12) is able to fit well some datasets of interest. Two suitable criteria are then adopted in order to discuss the goodness of fit, by taking into account also other popular growth curves, i.e. the Gompertz model (5) and the logistic model

23 $$\begin{aligned} N_L(t)=\frac{C}{1+\left( \frac{C-y}{y}\right) e^{-r\,t}}, \qquad t>0,\quad C,r,y>0. \end{aligned}$$

The considered data analytic examples are related to epidemiology and reliability contexts. Indeed, the three considered models are used to fit the dataset related to

- the active number and the total number of COVID-19 contagions from the 25th February to the 1st April 2020 in Iran and Italy (cf. [39] and [40], respectively);
- a set of Release #1 failure data coming from software products at Tandem Computers (cf. Wood [36]), reported in Table 11.

We use the nonlinear regression to fit the data, in particular the routine *lsqcurvefit* of Matlab$^{\textregistered }$, to solve nonlinear curve-fitting (data-fitting) problems in least-squares sense leading to the parameters estimation. Note that, we do not consider the Korf model (6) because for both datasets it does not perform a good fit, due to the fact that such a model has a slower growth when time increases. Moreover, we do not show the results related to the *DS* model (7) since it is a special case of the new model $N_{GPD}$, and thus the fitting is better for the latter one.

Aiming to identify the best fitting model, we use two procedures to estimate parameters and check the goodness. In particular, we compute the sum of squared residuals (SSR) with the routine *lsqcurvefit* and then we estimate the parameters by means of ISRP metric. Further we calculate the $d_2$-distance (in the euclidean norm) between ISRP and the constant parameter. We recall that the ISRP growth metric has been introduced recently in [5] aiming to determine the true growth curve that best fits the data (statistically). Indeed, such a metric provides an estimate of the rate parameter corresponding to the identified growth model in specific time intervals. The ISRP for the three considered models are respectively:

$$\begin{aligned}&ISRP_G= \frac{\beta }{e^{-\beta t}-e^{-\beta (t+\varDelta t)}} \log \left( \frac{N_G(t+\varDelta t)}{N_G(t)}\right) , \\&ISRP_L=\frac{1}{\varDelta t}\log \left[ \frac{\frac{c\,y}{C-y} \frac{1}{N_L(t)}-1}{\frac{c\,y}{C-y}\frac{1}{N_L(t+\varDelta t)}-1}\right] , \\&ISRP_{GPD}= \frac{1}{b\left[ \left( \frac{b}{at+b}\right) ^\frac{1}{a} -\left( \frac{b}{a(t+\varDelta t)+b}\right) ^\frac{1}{a}\right] } \log \left( \frac{N_{GPD}(t+\varDelta t)}{N_{GPD}(t)}\right) , \end{aligned}$$

with reference to the growth parameter $\alpha ,\,r,\,A$.

**Fig. 8:**
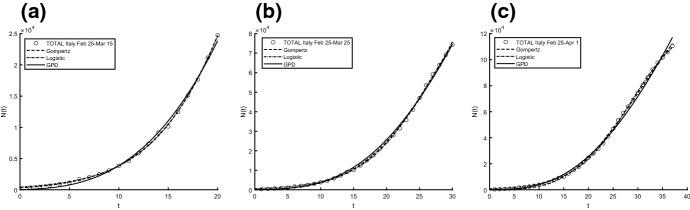
Interpolation of dataset (a1) for the total case in Italy under the Gompertz (5), logistic (23) and GPD (12) models. From left to right, **a** 25-th Feb/15-th March, **b** 25-th Feb/25-th March, **c** 25-th Feb/1-st April

**Fig. 9:**
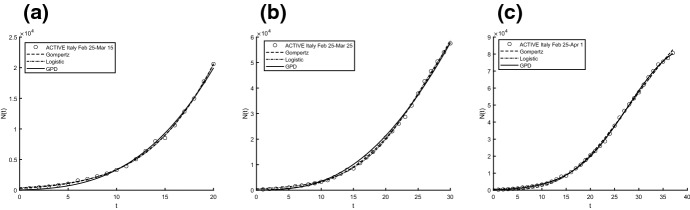
Interpolation of dataset (a1) for the active case in Italy under the Gompertz (5), logistic (23) and GPD (12) models. From left to right, the data for **a** 25-th Feb/15-th March, **b** 25-th Feb/25-th March, **c** 25-th Feb/1-st April

**Fig. 10:**
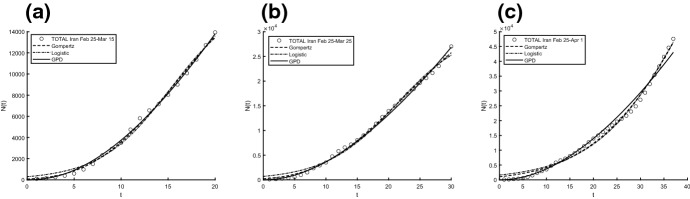
Interpolation of dataset (a1) for the total case in Iran under the Gompertz (5), logistic (23) and GPD (12) models. From left to right, **a** 25-th Feb/15-th March, **b** 25-th Feb/25-th March, **c** 25-th Feb/1-st April

**Fig. 11:**
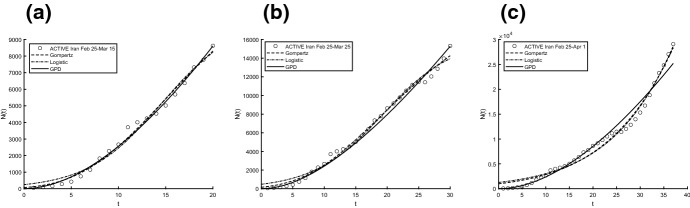
Interpolation of dataset (a1) for the active case in Iran under the Gompertz (5), logistic (23) and GPD (12) models. From left to right, **a** 25-th Feb/15-th March, **b** 25-th Feb/25-th March, **c** 25-th Feb/1-st April

**Fig. 12:**
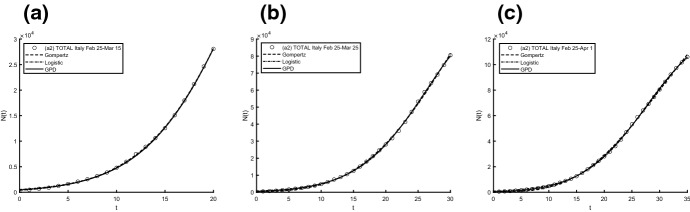
Interpolation of dataset (a2) for the total case in Italy under the Gompertz (5), logistic (23) and GPD (12) models. From left to right, **a** 25-th Feb/15-th March, **b** 25-th Feb/25-th March, **c** 25-th Feb/1-st April

**Fig. 13:**
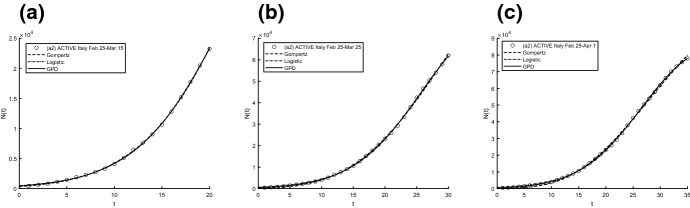
Interpolation of dataset (a2) for the active case in Italy under the Gompertz (5), logistic (23) and GPD (12) models. From left to right, the data for **a** 25-th Feb/15-th March, **b** 25-th Feb/25-th March, **c** 25-th Feb/1-st April

**Fig. 14:**
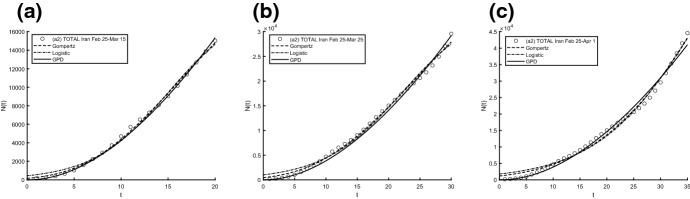
Interpolation of dataset (a2) for the total case in Iran under the Gompertz (5), logistic (23) and GPD (12) models. From left to right, **a** 25-th Feb/15-th March, **b** 25-th Feb/25-th March, **c** 25-th Feb/1-st April

**Fig. 15:**
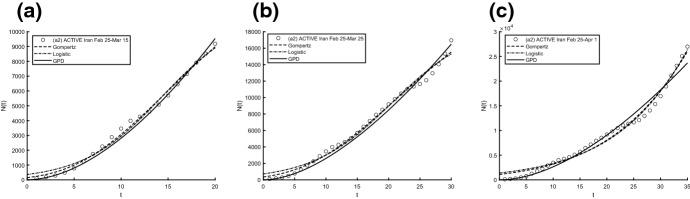
Interpolation of dataset (a2) for the active case in Iran under the Gompertz (5), logistic (23) and GPD (12) models. From left to right, **a** 25-th Feb/15-th March, **b** 25-th Feb/25-th March, **c** 25-th Feb/1-st April

**Fig. 16:**
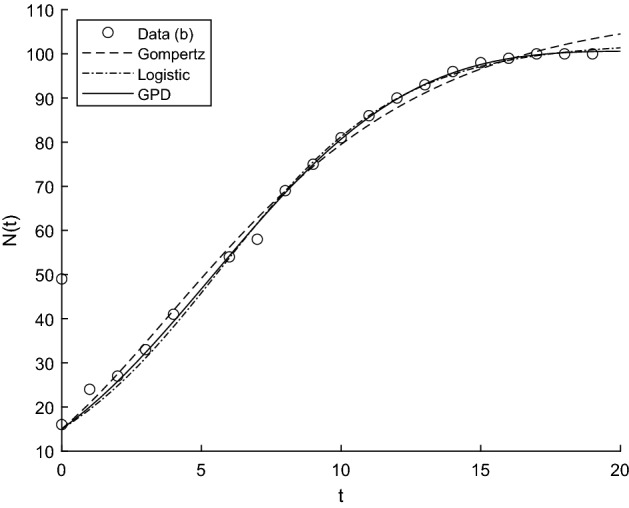
Interpolation of dataset (b)

- We consider two data collections: (a1) consists of COVID-19 data *n*(*i*) collected every day from February 25th to April 1st, 2020, whereas (a2) is formed by the mobile mean of data collected in 3 days, i.e. $data(i)=\frac{n(i)+n(i+1)+n(i+2)}{3}$, $i=1,2,\ldots ,35$. The analysis of the mobile mean is suggested by the fact that the reliability of data on infected by COVID-19 is subject to high variability caused by external factors, such as fluctuations in the availability of test devices and in the test response times.
  1. We show the interpolation of the dataset (a1), for three time intervals, where Figs. 8 and 9 refer to Italy and Figs. 10 and 11 refer to Iran. In all cases we have reasonably good fit. In Tables 1, 2 and 3, we report the SSR and the $d_2$-distance between ISRP and the constant parameter, for the given datasets (a1). The notation e+05, for example, means $\times 10^5$. In Table 4, Total and Active Coronavirus Cases in Italy (March 29-April 1) are compared with the prediction done with the GPD model (12). The parameters used for the prediction at *i*-th day come from the estimation performed until up to the $(i-1)$-th day, with the routine *lsqcurvefit* of Matlab$^{\textregistered }$. The same analysis is shown in Table 5 for Iran, whose Total and Active Coronavirus Cases (March 25-March 29) are compared with the prediction done with the GPD model (12).
  2. We show the interpolation of the dataset (a2), for three time intervals, for Italy in Figs. 12 and 13, and for Iran in Figs. 14 and 15. In all cases we have reasonably good fit. In Tables 6, 7 and 8, we report the SSR and the $d_2$-distance between ISRP and the constant parameter, for the given dataset (a2). In Table 9, Total and Active Coronavirus Cases in Italy (March 28-March 31) are compared with the prediction done with the GPD model (12). The parameters used for the prediction at *i*-th day come from the estimation performed up to the $(i-1)$-th day. The same analysis is performed in Table 10, where Total and Active Coronavirus Cases in Iran (March 28-March 31) are compared with the prediction done with the GPD model (12).
  We report in Tables 11 and 12 the estimates of the parameters for Figs. 8, 9, 10, 11, 12, 13, 14 and 15, and datasets (a1) and (a2), respectively, obtained by means of the routine *lsqcurvefit* of Matlab$^{\textregistered }$. By comparing the *SSR* and $d_2$ indexes of the two performed analysis, i.e. cases (a1) and (a2), we observe that (a2) provides better results. Indeed (a2) takes into account the running average of the data over 3 days. We find that according to the different intervals of time, and the adopted criteria (*SSR* and $d_2$), there is a variety in the detection of the best fitting model. Nevertheless, in the most of cases our proposed GPD model gives the best fitting.
- We show in Table 13 the considered data, and in Fig. 16 we interpolate the data with the Gompertz, the Logistic and the GPD models. In Table 13 we report also the SSR and the $d_2$-distance between ISRP and the constant parameters $A,\,\alpha ,\,r$, respectively. In these cases, we can observe that the GPD model fits better than the others.

**Table 1:** The SSR and $d_2$-distance between ISRP and the constant parameter, for data (a1) in the time interval 25-th Feb/15-th March

	GPD	Gompertz	Logistic
Total Italy			
*SSR*	7.96e+06	5.28e+05	8.15e+05
$d_2$	0.51	0.27	0.92
Active Italy			
*SSR*	3.92e+06	6.92e+05	8.24e+05
$d_2$	0.51	0.33	0.92
Total Iran			
*SSR*	8.61e+05	1.25e+06	2.94e+06
$d_2$	0.93	3.01	1.88
Active Iran			
*SSR*	1.38e+05	4.18e+05	1.42e+05
$d_2$	2.42	4.06	2.23

**Table 2:** The SSR and $d_2$-distance between ISRP and the constant parameter, for data (a1) in the time interval 25-th Feb/25-th March

	GPD	Gompertz	Logistic
Total Italy			
*SSR*	9.35e+07	1.26e+07	7.32e+06
$d_2$	0.29	0.42	1.03
Active Italy			
*SSR*	8.11e+07	9.28e+06	5.94e+06
$d_2$	0.34	0.53	1.02
Total Iran			
*SSR*	2.75e+06	1.48e+06	4.63e+06
$d_2$	1.13	0.85	0.94
Active Iran			
*SSR*	3.29e+06	1.55e+06	3.18e+06
$d_2$	2.14	2.11	1.15

**Table 3:** The SSR and $d_2$-distance between ISRP and the constant parameter, for data (a1) in the time interval 25-th Feb/1-st April

	GPD	Gompertz	Logistic
Total Italy			
*SSR*	9.01+06	3.53e+07	1.74e+07
$d_2$	0.31	0.84	3.22
Active Italy			
*SSR*	7.47e+06	3.35e+07	1.05e+07
$d_2$	0.42	1.11	1.08
Total Iran			
*SSR*	6.97e+07	3.31e+07	3.61e+07
$d_2$	1.56	1.06	0.52
Active Iran			
*SSR*	6.40e+07	3.30e+07	3.60+07
$d_2$	1.56	0.52	1.06

**Table 4:** Total and active coronavirus cases in Italy versus the prediction done with the GPD model based on data (a1)

Day	29-th March	30-th March	31-th March	1-st April
Predicted total	96,789	102,153	106,058	109,279
Real total	97,689	101,739	105,792	110,574
Predicted active	75,748	76,713	78,268	80,375
Real active	73,880	75,528	77,635	80,672

**Table 5:** Total and active coronavirus cases in Iran versus the prediction done with the GPD model based on data (a1)

Day	29-th March	30-th March	31-th March	1-st April
Predicted total	37,260	39,870	43,477	46,974
Real total	38,309	41,495	44,605	47,593
Predicted active	21,754	23,481	25,128	27,094
Real active	23,278	24,827	27,051	29,084

**Table 6:** The SSR and the ISRP metric for data (a2) in the time interval 25-th Feb/15-th March

	GPD	Gompertz	Logistic
Total Italy			
*SSR*	2.1e+05	2.84e+05	3.09e+05
$d_2$	0.12	0.11	0.94
Active Italy			
*SSR*	1.36e+06	3.11e+05	2.33e+05
$d_2$	0.15	0.15	0.93
Total Iran			
*SSR*	6.91e+05	9.42e+05	2.68e+06
$d_2$	0.72	0.42	1.06
Active Iran			
*SSR*	5.21e+05	9.83e+05	1.91e+06
$d_2$	0.77	0.63	1.01

**Table 7:** The SSR and the ISRP metric for data (a2) in the time interval 25-th Feb/25-th March

	GPD	Gompertz	Logistic
Total Italy			
*SSR*	3.17e+06	1.06e+07	5.03e+06
$d_2$	0.15	0.29	1.02
Active Italy			
*SSR*	2.4e+06	7.28e+06	3.81e+06
$d_2$	0.17	0.34	1.01
Total Iran			
*SSR*	1.06e+07	8.97e+06	1.99e+07
$d_2$	1.05	0.7	0.84
Active Iran			
*SSR*	4.01e+06	6.23e+06	1.05e+07
$d_2$	0.96	0.95	0.83

**Table 8:** The SSR and the ISRP metric for data (a2) in the time interval 25-th Feb/1-st April

	GPD	Gompertz	Logistic
Total Italy			
*SSR*	4.82+06	2.5e+07	1.21e+07
$d_2$	0.14	1.04	0.58
Active Italy			
*SSR*	3.30e+06	2.23e+07	6.33e+06
$d_2$	0.18	0.7	1.05
Total Iran			
*SSR*	5.85e+07	4.05e+07	5.71e+07
$d_2$	1.2	0.78	0.62
Active Iran			
*SSR*	3.84e+07	2.84e+07	3.15+07
$d_2$	0.86	0.87	0.51

**Table 9:** Total and active coronavirus cases in Italy versus the prediction done with the GPD model based on data (a2)

Day	28-th March	29-th March	30-th March	31-th March
Predicted total	93,332	98,091	102,302	106,001
Real total	92,472	97,689	101,739	105,792
Predicted active	71,690	74,328	76,954	77,082
Real active	70,065	73,880	75,528	77,635

**Table 10:** Total and active coronavirus cases in Iran versus the prediction done with the GPD model based on data (a2)

Day	28-th March	29-th March	30-th March	31-th March
Predicted total	36,827	39,694	42,775	45,031
Real total	35,408	38,309	41,495	44,605
Predicted active	20,366	21,734	22,628	24,787
Real active	21,212	23,278	24,827	27,051

**Table 11:** The estimates of the parameters for Figs. 8, 9, 10 and 11 and dataset (a1)

Model parameters	GPD	Gompertz	Logistic
	$y,\;A,\;a,\;b$	$y,\;\alpha ,\;\beta$	$y,\;r,\;C$
Figure 8			
(a)	$33.86,\;0.99,\;5.54 ,\;24.94$	$312.57,\;0.29,\;0.03$	$441.71,\;0.22,\;108083$
(b)	$68.21,\;0.61,\;0.64,\;16.11$	$127.05,\;0.43,\;0.05$	$595.98,\;0.19,\;335555$
(c)	$37.05,\;0.84,\;1.36,\;14.12$	$31.92,\;0.62,\;0.07$	$763.65,\;0.18,\; 308078$
Figure 9			
(a)	$48.46,\;0.76,\;2.61,\;20.61$	$287.61,\;0.28,\;0.03$	$397.25,\;0.21,\; 75702$
(b)	$19.83,\;1.06,\;1.83,\;12.43$	$114.12,\;0.43,\;0.05$	$531.59,\;0.19,\;299301$
(c)	$266.05,\;0.29,\;-0.49,\;19.75$	$21.95,\;0.67,\;0.07$	$620.14,\;0.18,\;250126$
Figure 10			
(a)	$0.78,\;7.45,\;5.64,\;2.69$	$72.12,\;0.63,\;0.1$	$295.58,\;0.26,\;22478$
(b)	$2.09,\;5.81,\;7.36,\;3.82$	$215.37,\;0.4,\;0.07$	$696.55,\;0.17,\;48793$
(c)	$0.44,\;12.29,\;11.94,\;2.68$	$1033.1,\;0.2,\;0.01$	$1615.1,\;0.1,\;35572700$
Figure 11			
(a)	$1.69,\;6.67,\;8.97,\;3.6$	$70.59,\;0.59,\;0.11$	$247.51,\;0.25,\; 15931$
(b)	$1.4,\;6.74,\;7.11,\;3.05$	$160.89,\;0.4,\;0.08$	$478.46,\;0.17,\;14322$
(c)	$1.19,\;7.27,\;11.54,\;4.12$	$992.7,\;0.11,\;0.01$	$1265,\;0.1,\;27863500$

**Table 12:** The estimates of the parameters for Figs. 12, 13, 14 and 15 and dataset (a2)

Model parameters	GPD	Gompertz	Logistic
	$y,\;A,\;a,\;b$	$y,\;\alpha ,\;\beta$	$y,\;r,\;C$
Figure 12			
(a)	$450.76,\;0.25,\;-0.74,\;17.65$	$375.96,\;0.31,\;0.03$	$547.59,\;0.22,\;133991$
(b)	$486.89,\;0.25,\;-0.61,\;20.42$	$174.97,\;0.42,\;0.05$	$767.64,\;0.19,\;431844$
(c)	$384,\;0.29,\;-0.47,\;19.74$	$77.57,\;0.54,\;0.06$	$911.76,\;0.18,\; 367830$
Figure 13			
(a)	$446.41,\;0.24,\;-0.78,\;17.12$	$341.83,\;0.3,\;0.03$	$490.2,\;0.22,\;119948$
(b)	$433.66,\;0.25,\;-0.59,\;19.83$	$159.42,\;0.42,\;0.05$	$681.83,\;0.19,\;383891$
(c)	$375.33,\;0.27,\;-0.50,\;19.39$	$60.42,\;0.56,\;0.07$	$758.03,\;0.18,\;305811$
Figure 14			
(a)	$0.67,\;11.11,\;9.73,\;2.49$	$157.82,\;0.52,\;0.1$	$438.91,\;0.24,\;23963$
(b)	$1.37,\;7.87,\;10.57,\;3.67$	$437.34,\;0.31,\;0.06$	$1001.4,\;0.2,\;48584400$
(c)	$0.45,\;12.75,\;12.8,\;2.71$	$1157.9,\;0.2,\;0.01$	$1759.6,\;0.1,\;38757800$
Figure 15			
(a)	$1.53,\;8.18,\;13.38,\;3.93$	$150.05,\;0.48,\;0.10$	$360.52,\;0.23,\; 16661$
(b)	$1.88,\;7.24,\;10.75,\;3.67$	$346.72,\;0.29,\;0.06$	$716.36,\;0.15,\;30460$
(c)	$0.77,\;10.7,\;13.54,\;3.11$	$1091.6,\;0.1,\;0.01$	$1389.6,\;0.1,\;3060800$

**Table 13:** Data (b): Tandem Computers software failure Data; SSR and $d_2$-distance between ISRP and the constant parameter

Testing times (weeks)	1	2	3	4	5	6	7	8	9	10
Defects found	16	24	27	33	41	49	54	58	69	75
Testing times (weeks)	11	12	13	14	15	16	17	18	19	20
Defects found	81	86	90	93	96	98	99	100	100	100

Data (b)	GPD	Gompertz	Logistic							
*SSR*	39.79	79.34	63.55							
$d_2$	0.55	0.71	1.32							

## Analysis of a special inhomogeneous linear birth-death process

Birth-death processes are largely used to model random evolution. See Callaert and Keilson [6, 7] for the spectral structure of such processes. In this section, we consider a stochastic counterpart of the growth model introduced in (12) by studying an evolutionary model based on a birth-death process. We will consider a continuous-time Markov chain having an infinite state-space to mimics the growth curve (12) which can reach any high level when *A* is large. In particular, we consider a time-inhomogeneous linear birth-death process $\left\{ X(t);\,t \ge 0\right\}$ having state space ${\mathbb {N}}_0$, and the absorbing endpoint 0. Denoting by

$$\begin{aligned} q_{i,j}(t)=\lim _{h \rightarrow 0} \frac{1}{h}\, {\mathbb {P}}[X(t+h)=j \,|\,X(t)=i], \qquad i,j \in {\mathbb {N}}_0 \end{aligned}$$

the time-dependent transition rates of *X*(*t*), we assume that

24 $$\begin{aligned} q_{i,j}(t)=\left\{ \begin{array}{ll} i\,\lambda (t), &{} \quad j=i+1, \; i\in {\mathbb {N}}_0 \qquad \hbox {(birth rate)}, \\ i\,\mu (t), &{} \quad j=i-1, \; i \in {\mathbb {N}} \qquad \hbox {(death rate)}, \end{array}\right. \end{aligned}$$

where $\lambda (t)$ and $\mu (t)$ are positive functions, integrable on (0, *t*) for any finite $t> 0$. Note that the rates (24) are linear in *i*, hence the intensities of new births and deaths are proportional to the population size at the current time, and to the time-dependent functions $\lambda (t)$ and $\mu (t)$, respectively. Clearly, $\lambda (t)$ and $\mu (t)$ represent the individual birth rate and death rate at time *t*, respectively. We denote with $P_{y,x}(t)$ the transition probability of *X*(*t*), for all $t\ge 0$, $x\in {\mathbb {N}}_0$ and $y \in {\mathbb {N}}$, i.e. the probability that the population, starting from *y*, reaches level *x* at time *t*. Note that, we consider $X(0)=y\in {\mathbb {N}}$ as for the growth model (12) the initial state is positive. Taking into account the results obtained in [12], the process *X*(*t*) with rates (24) has transition probabilities, for $y\in {\mathbb {N}}$,

25 $$\begin{aligned} P_{y,0}(t)= & {} \left( 1-\frac{1}{\psi +\phi }\right) ^{ y}, \end{aligned}$$

26 $$\begin{aligned} P_{y,x}(t)= & {} \left( \frac{\phi }{\psi +\phi }\right) ^{ x} \, \sum _{i=0}^{m} {y \atopwithdelims ()i}{y+x-i-1 \atopwithdelims ()y-1} (\phi ^{-1}-1)^{i}\left( 1-\frac{1}{\psi +\phi }\right) ^{ y-i},\nonumber \\&\quad \qquad x\in {\mathbb {N}}, \end{aligned}$$

with $m=\min \left\{ y,x\right\}$, where $\psi =\psi (t)$ and $\phi =\phi (t)$ are given by:

27 $$\begin{aligned} \psi (t)=\exp {\left\{ -\int _0^t [\lambda (\tau )-\mu (\tau )]\,\mathrm{d}\tau \right\} }, \qquad \phi (t)=\int _0^t \lambda (\tau ) \psi (\tau ) \,\mathrm{d}\tau . \end{aligned}$$

Moreover, the conditional mean $E_y(t)={\mathbb {E}}[X(t)|X(0)=y]$ and the conditional variance $V_y(t)=\mathrm{Var}[X(t)|X(0)=y]$ of *X*(*t*) are respectively

28 $$\begin{aligned} E_y(t)=\frac{y}{\psi (t)}, \qquad V_y(t)=y \,\frac{[\psi (t)+2 \phi (t)-1]}{\psi ^2(t)}, \qquad t\ge 0. \end{aligned}$$

Recalling the birth and death rates (24), the population mean satisfies the following differential equation:

29 $$\begin{aligned} \frac{d \, E_y(t)}{dt}=\xi (t) E_y(t),\qquad t>0, \end{aligned}$$

where $\xi (t)$ is the net growth rate *per capita* of individuals, i.e.

30 $$\begin{aligned} \xi (t)=\lambda (t)- \mu (t), \qquad t\ge 0. \end{aligned}$$

Hence, by noting that equations (29) and (4) have the same form, we get that the mean of the process *X*(*t*) is equal to the growth curve proposed in (12), under the assumptions (11) and (30). Some properties of $E_y(t)$ and $V_y(t)$ are provided in Table 4 of [12]. Finally, we observe that $P_{y,0}(t)$ is the probability that the population reaches the extinction prior to time *t*, being 0 an absorbing endpoint. In particular, from (25) the probability of ultimate extinction, conditional by the initial size *y*, is

31 $$\begin{aligned} \pi _{y,0}:=\lim _{t\rightarrow \infty } P_{y,0}(t) =\left( 1-\frac{1}{\tilde{\psi }+\tilde{\phi }}\right) ^{ y}, \qquad y\in {\mathbb {N}}, \end{aligned}$$

where

32 $$\begin{aligned} \tilde{\psi }=\lim _{t \rightarrow \infty } \psi (t), \quad \tilde{\phi }=\lim _{t \rightarrow \infty } \phi (t), \quad \tilde{\lambda }=\lim _{t \rightarrow \infty } \int _0^t \lambda (\tau ) \,\mathrm{d}\tau , \quad \tilde{\mu }=\lim _{t \rightarrow \infty } \int _0^t \mu (\tau ) \,\mathrm{d}\tau . \end{aligned}$$

Obviously, if $\tilde{\mu }-\tilde{\lambda }=\infty$ or $\tilde{\phi }=\infty$, then $\pi _{y,0}=1$, i.e. the ultimate extinction is certain.

### Analysis of a special case

In the following we assume that $a>0$, that corresponds to the case (i) of the analysis performed in Sect. 2. This choice leads to a process *X*(*t*) having an increasing behavior and tending to a carrying capacity. As observed before, the conditional mean of *X*(*t*) verifies the same law of the growth model (12), under Eqs. (11) and (30). In this section, we focus our attention on this special case.

#### Proposition 1

The linear birth-death process *X*(*t*) with rates specified in (24) has conditional mean

33 $$\begin{aligned} E_y(t)= y \exp {\left\{ Ab\left[ 1-\left( \frac{b}{at +b}\right) ^{ \frac{1}{a}}\right] \right\} }, \qquad t\ge 0 \end{aligned}$$

if and only if

34 $$\begin{aligned} \lambda (t)-\mu (t)=A \left( \frac{b}{at+b}\right) ^{ \frac{1}{a}+1}, \qquad t\ge 0. \end{aligned}$$

#### Proof

We substitute the first equation of (27) in (28) and we obtain

$$\begin{aligned} E_y(t)=y \exp {\left\{ \int _0^t [\lambda (\tau )-\mu (\tau )] \,\mathrm{d}\tau \right\} }, \qquad t\ge 0. \end{aligned}$$

Hence, the expression (33) holds if and only if

35 $$\begin{aligned} \int _0^t [\lambda (\tau )-\mu (\tau )]\,\mathrm{d}\tau =Ab\left[ 1-\left( \frac{b}{at+b}\right) ^{ \frac{1}{a}}\right] , \qquad t\ge 0, \end{aligned}$$

that is equivalent to (34). $\square$

We now investigate on the process *X*(*t*) taking into account the relation (34). In this case, we have $\lambda (t)>\mu (t)$, and thus the net growth rate $\xi (t)$ defined in (30) is decreasing and tends to 0 following a power law. Therefore, a population whose conditional mean has a *S*-shape is well described in this case. Moreover, for (27), the mean $E_y(t)$ is identical to the curve *N*(*t*) given in (12). Hence, due to (34), from (35) one obtains $\tilde{\lambda }-\tilde{\mu }=Ab$, with $\tilde{\lambda }$ and $\tilde{\mu }$ defined in (32). From the results shown in Table 4 of [12], we have that $E_y(t)$ is strictly increasing, and it tends to the carrying capacity defined in (14), i.e. $\lim _{t\rightarrow \infty } E_y(t)=y e^{Ab} \equiv C$. Moreover, we have

36 $$\begin{aligned} \psi (t)=\exp {\left\{ -Ab\left[ 1-\left( \frac{b}{at+b} \right) ^{ \frac{1}{a}}\right] \right\} }, \qquad t\ge 0, \end{aligned}$$

with $\tilde{\psi }=e^{-Ab}$, so that $V_y(t)$ is strictly increasing in *t*.

#### Example 1

**Fig. 17:**
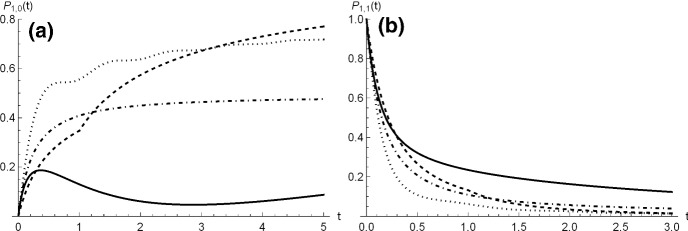
Probabilities $$P_{1,0}(t)$$ and $$P_{1,1}(t)$$ for the cases specified in Table 13, where $$c=2.00001$$ in all cases, with **a** (full line), **b** $$d=1$$, $$ t_0=1$$ (dashed line), **c** $$d=2$$, $$Q=1$$ (dotted line), **d** (dot-dashed line)

Under the validity of Eq. (34), we now consider various choices of the function $\mu (t)$ listed in Table 14. The transition probabilities (25) and (26) are plotted in Fig. 17 for $y=1$, $x=0$ and $x=1$, and for some choices of the parameters. In Table 14 the last column is dedicated to the asymptotic absorption probability (31).

- We consider $\mu (t) = c$, with $c>0$, i.e. the individual death rate of the populations is constant. From (27) one has, for $t>0$,
  37 $$\begin{aligned} \phi _a(t)=c\,b\,e^{-Ab}(-Ab)^a\,\gamma _a(t)+1 -\exp {\left\{ -Ab\left[ 1-\left( \frac{b}{at+b}\right) ^{\frac{1}{a}}\right] \right\} }, \end{aligned}$$
  with $\gamma _a(t)=\varGamma \left( -a,-Ab\left( \frac{b}{at+b} \right) ^{\frac{1}{a}}\right) -\varGamma \left( -a,-Ab\right)$, where $\varGamma (\cdot ,\cdot )$ is the upper incomplete Gamma function. The expression of the variance follows from (28), where $\psi$ is given in (36).
- We consider $\mu (t)=c+d\,(t-t_0) {\mathbb {I}}_{ \{t\ge t_0\} }$, with $c>0$, $d>0$ and $t_0>0$, where ${\mathbb {I}}_A$ is the indicator function, such that ${\mathbb {I}}_A=1$ if *A* is true, and 0 otherwise. In this case the individual death rate is constant until time $t_0$ and is linear increasing afterwards, for instance due to worsening of the environmental or individual conditions. From (27) one has
  $$\begin{aligned} \phi _b(t)=\phi _a(t)+d\,e^{Ab}\,b\,(-Ab)^a\left( \gamma _{b2}(t)-\gamma _{b1}(t)\right) , \end{aligned}$$
  where $\phi _a(t)$ is defined in (37) and
  $$\begin{aligned} \gamma _{b1}(t)= & {} \varGamma \left[ -a,k(t)\right] -\varGamma \left[ -a,k(t_0)\right] ,\\ \gamma _{b2}(t)= & {} (-Ab)^a\left\{ \varGamma \left[ -2a,k(t)\right] -\varGamma \left[ -2a,k(t_0)\right] \right\} +\varGamma \left[ -a,k(t_0)\right] \\&-\varGamma \left[ -a,k(t)\right] , \end{aligned}$$
  with $k(t)=-Ab\left( \frac{b}{at+b}\right) ^{ \frac{1}{a}}$. The expression of the variance follows from (28).
- Let $\mu (t)=c+d \sin \left( \frac{2 \pi }{Q}t \right)$, where $Q>0$ and $c> |d|>0$. This case describes populations subject to individual sinusoidal death rate, due to i.e. periodic increase and decrease of mortality. The function (27) can be expressed in integral form, and also the variance (28).
- Let $\mu (t)= c \,\left( \frac{b}{at+b} \right) ^{\frac{1}{a}+1}$, with $c>0$; in this case the individual birth and death rates are proportional; the function (27) becomes
  $$\begin{aligned} \phi _d(t)=\left( \frac{c}{a}+1\right) \left( 1-\exp {\left\{ -Ab \left[ 1-\left( \frac{b}{at+b}\right) ^{ \frac{1}{a}}\right] \right\} }\right) , \end{aligned}$$
  and the variance is obtained from (28).

**Fig. 18:**
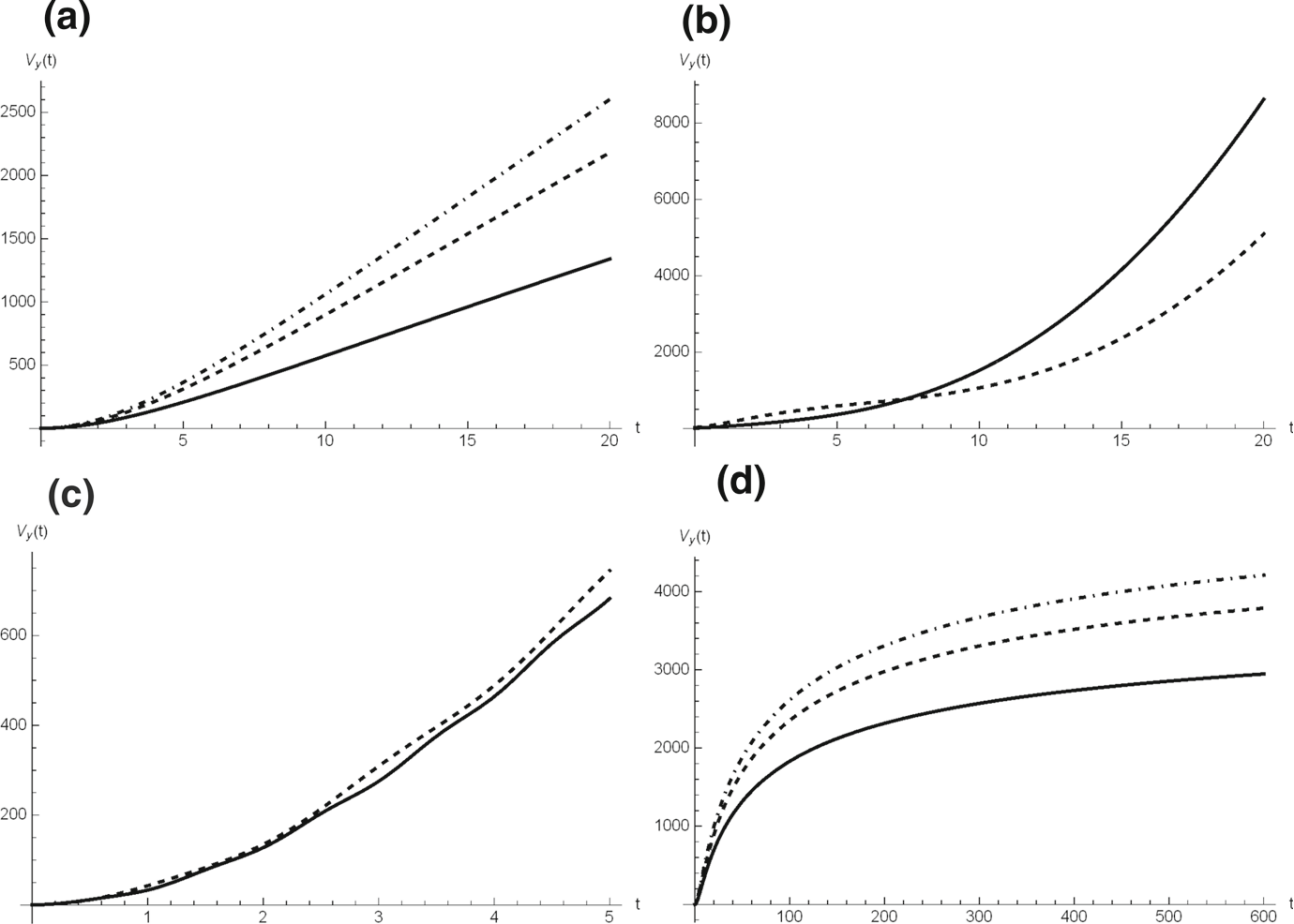
For $y=1,\,A=2,\, a,b=2$, the variance $V_y(t)$ is plotted for the cases of Table 13. In case (**a**): $c = 0.4,\, 0.8,\,1$ (from bottom to top). In case (**b**): $c=1$, $d=1$, $t_0=5$ (solid line) and $t_0=10$ (dashed line). In case (**c**): $c=2.00001$, $d=2$, $Q=1$ (solid line) and $Q=2$ (dashed line). In case (**d**): $c=0.4,\,0.8,\,1$ (from bottom to top)

In the first three cases of Table 14 one has $\tilde{\psi }=+\infty$ because $\tilde{\mu }=+\infty$, therefore $\pi _{y,0}=1$, i.e. the ultimate extinction is certain. On the contrary, $\tilde{\mu }<+\infty$ in case (d), and thus $\tilde{\psi }<+\infty$, so that $\pi _{y,0}<1$. Moreover, we note that the variance diverges as $t\rightarrow \infty$ for cases (a), (b) and (c), whereas it converges to a constant in case (d). In Fig. 18 we show some plots of $V_y(t)$, depending on $\mu (t)$.

**Table 14:** Some choices of $\mu (t)$ and the corresponding extinction probabilities (31)

Case	$\mu (t)$	$\pi _{y,0}$
(a)	*c*	1
(b)	$c+d \,(t-t_0){\mathbb {I}}_{ \{t\ge t_0\} }$	1
(c)	$c+d\, \sin \left( \frac{2\pi }{Q}t\right)$	1
(d)	$c \,\left( \frac{b}{at+b}\right) ^{\frac{1}{a}+1}$	$\left[ \frac{\frac{c}{A} \left( 1-e^{-Ab}\right) }{\frac{c}{A}\left( 1-e^{-Ab}\right) +1}\right] ^y$

Parameters *c*, *d*, *Q* and $t_0$ are positive constants

## Analysis of a special time-inhomogeneous linear birth process

In the previous section, we considered a birth-death process with conditional mean identical to the growth curve (12). Nevertheless the sample paths of that process may not reflect the behavior of the growth curve since they can be absorbed at zero (see the probabilities given in Eqs. (25) and (31)). In order to deal with a stochastic process more suitable to describe a growth phenomenon, we remove the possibility of downward jumps by assuming that $\mu (t)\equiv 0$ in Eq. (24). This leads to a time-inhomogeneous linear birth process $\left\{ X(t);\,t \ge 0\right\}$, that possesses non-decreasing sample paths, and is characterized by time-dependent birth rates

38 $$\begin{aligned} q_{k,k+1}(t)=k\,\lambda (t), \qquad k \in {\mathcal {S}}. \end{aligned}$$

Here, ${\mathcal {S}}=\{y,y+1,\ldots \}$ is the state space and $X(0)=y\in {\mathbb {N}}$ is the initial state. Clearly, the function $\lambda (t)$ is continuous, positive and integrable on (0, *t*), for any $t>0$. As usual, we denote with $P_{y,x}(t)={\mathbb {P}}[X(t) =x\,|\,X(0)=y]$ the transition probabilities of *X*(*t*). For $y\in {\mathbb {N}}$ and $x\in {\mathcal {S}}$, one has (see, for example, [28]):

39 $$\begin{aligned} P_{y,x}(t)={x-1\atopwithdelims ()y-1} e^{-y \varLambda (t)} \left( 1-e^{-\varLambda (t)}\right) ^{x-y},\qquad t\ge 0, \end{aligned}$$

where

40 $$\begin{aligned} \varLambda (t)=\int _0^t \lambda (\tau )\,\mathrm{d}\tau , \qquad t\ge 0 \end{aligned}$$

is the individual time-dependent cumulative birth intensity. By recalling Proposition 3 of Di Crescenzo and Spina [12] and the time-dependent growth rate (11), for $a>0$, we have that the linear birth process with rates (38) has conditional mean

41 $$\begin{aligned} E_y(t)= y\,\exp \left\{ Ab\left[ 1-\left( \frac{b}{at+b} \right) ^{1/a}\right] \right\} , \qquad t\ge 0 \end{aligned}$$

if and only if

42 $$\begin{aligned} \lambda (t)=A \left( \frac{b}{at+b}\right) ^{\frac{1}{a}+1}, \qquad t\ge 0. \end{aligned}$$

This condition allows the conditional mean of the time-inhomogeneous birth process $\left\{ X(t);\,t \ge 0\right\}$ to be equal to the growth curve given in Eq. (12). Moreover, from (40) and (42) we have

43 $$\begin{aligned} \varLambda (t)=Ab\left[ 1-\left( \frac{b}{at+b}\right) ^{1/a}\right] , \end{aligned}$$

and

44 $$\begin{aligned} \varLambda (t)\quad {\mathop {\rightarrow }\limits ^{t\rightarrow +\infty }} \quad Ab=\int _0^\infty \lambda (\tau )\,\mathrm{d}\tau . \end{aligned}$$

**Fig. 19:**
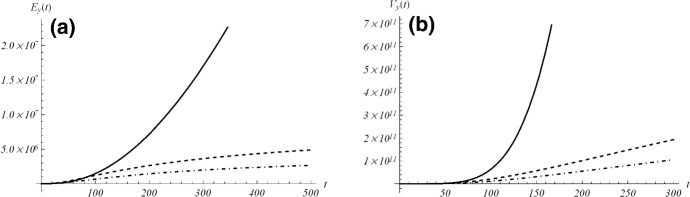
The mean (45), on the left, and the variance (46), on the right, are plotted for the estimated values of Fig. 8 (reported in Table 11) for model GPD, from top to bottom for the cases (**a**), (**b**) and (**c**). On the left, one has $E_y(\infty )=1.74\times 10^{12},\,9.62\times 10^6,\,5.23\times 10^6$; on the right, it is $V_y(\infty )=9.21\times 10^{22},\,1.36 \times 10^{12},\,7.41\times 10^{11}$, for the cases (**a**), (**b**) and (**c**), respectively

This property reflects that the births occur with decreasing time inputs, which is typical for environments with limited resources. Indeed, under condition $a > 0$ the process $\left\{ X(t);\,t \ge 0\right\}$ reaches a mean saturation level. This is confirmed by the fact that the conditional mean (41) and the related variance are strictly increasing, with finite limits

45 $$\begin{aligned} E_y(t)= & {} y\,e^{\varLambda (t)} \quad {\mathop {\rightarrow }\limits ^{t\rightarrow +\infty }} \quad E_y(\infty ) \equiv ye^{Ab}, \end{aligned}$$

46 $$\begin{aligned} V_y(t)= & {} ye^{\varLambda (t)} \left( e^{\varLambda (t)}-1\right) \quad {\mathop {\rightarrow }\limits ^{t\rightarrow +\infty }} \quad V_y(\infty )\equiv ye^{Ab}\left( e^{Ab}-1\right) , \end{aligned}$$

for $\varLambda (t)$ given in (43). In Fig. 19 the mean (45) and the variance (46) are plotted for suitable choices of the parameters taken from the data considered in Sect. 4. We remark that the mean and variance exhibit a greater growth for the parameters considered in the time interval (a), that refers to a case in which the lockdown effects were not yet prevailing.

For this process, we also obtain the *Fano factor* (i.e. the *index of dispersion*, defined as the variance over the mean). Indeed, due to Eqs. (45) and (46), the following results hold:

47 $$\begin{aligned} D_y(t):=\frac{V_y(t)}{E_y(t)}=e^{\varLambda (t)}-1 \quad {\mathop {\rightarrow }\limits ^{t\rightarrow +\infty }} \quad e^{Ab}-1. \end{aligned}$$

We thus obtain that $D_y(t)$ is monotonic increasing in *t*, with $D_y(0)=0$. Moreover, by analysing (47), we note that:

(i) If $0<A<\frac{1}{b}\log 2$ then *X*(*t*) is underdispersed, i.e. $D_y(t)<1$ for all $t\ge 0$; in this case the occurrence of births is more regular than a Poisson process.
(ii) If $A>\frac{1}{b}\log 2$ then *X*(*t*) is underdispersed for $t<t_*$, and overdispersed for $t>t_*$, where
  $$\begin{aligned} t_*:=\frac{b}{a} \left[ \left( 1-\frac{\log 2}{Ab}\right) ^{-a}-1\right] ; \end{aligned}$$
  therefore there is more irregularity in the distribution of births with respect to a Poisson process, for large times.

Now we consider the *coefficient of variation* of *X*(*t*), that from (45) and (46) results:

$$\begin{aligned} \sigma _y(t)= \frac{\sqrt{V_y(t)}}{E_y(t)} =y^{-1/2} \sqrt{1- e^{-\varLambda (t)}}, \qquad t\ge 0, \end{aligned}$$

where $\varLambda (t)$ is given in (43); so $\sigma _y(t)$ is increasing in *t*, in *A*, *a* and *b*. Moreover, the following limiting behaviors are obtained:

$$\begin{aligned}&\displaystyle \sigma _y(t) \quad {\mathop {\rightarrow }\limits ^{t\rightarrow +\infty }} \quad y^{-1/2} \sqrt{1- e^{-Ab}},\\&\displaystyle \lim _{A \rightarrow 0} \sigma _y(t)=0, \qquad \lim _{A \rightarrow +\infty } \sigma _y(t)=y^{-1/2},\\&\displaystyle \lim _{a \rightarrow 0} \sigma _y(t)=y^{-1/2}\sqrt{1-e^{-Abe^{-t/b}}}, \qquad \lim _{a \rightarrow + \infty } \sigma _y(t)=y^{-1/2}\sqrt{1-e^{-Ab}},\\&\displaystyle \lim _{b \rightarrow 0} \sigma _y(t)=0, \qquad \lim _{b \rightarrow +\infty } \sigma _y(t)=y^{-1/2}. \end{aligned}$$

Therefore, when $A \rightarrow 0$ or $b \rightarrow 0$ there is a good correspondence between the deterministic law (12) and the stochastic process with birth rates (38). Clearly, if $A \rightarrow 0$ or $b \rightarrow 0$, then $\lambda (t) \rightarrow 0$. This yields that for a low birth rate the two models exhibit a good agreement.

It is worth mentioning that the knowledge of the transition probabilities (39) allows to perform a stochastic comparison for the birth processes considered in this section. To this aim we recall the following notion: Given two discrete random variables $X_i$, $i=1,2$, we say that $X_1$ is smaller than $X_2$ in the likelihood ratio order (denoted by $X_1 \le _\mathrm{lr} X_2$) if ${\mathbb {P}}(X_2=x)/{\mathbb {P}}(X_1=x)$ increases in *x* over the union of the supports of $X_1$ and $X_2$ (see Section 1.C of [30]). Then, in analogy with Remark 3 we are able to provide the following result.

### Remark 4

From (10) it is not hard to see that for the GPD distribution one has that $\int _{0}^t F(\tau )\,\mathrm{d}\tau$ is increasing in $a\in (-1,0)\cup (0,+\infty )$ and is decreasing in $b\in (0,+\infty )$ for all $t\ge 0$. Hence, denoting by $N_{GPD, i}(t)$ the growth model (12) characterized by parameters $y_i, A_i,a_i,b_i$, $i=1,2$, due to Remark 2 we have that if $y_1 \le y_2$, $A_1 \le A_2$, $a_1 \ge a_2$ and $b_1 \le b_2$, then

$$\begin{aligned} N_{GPD, 1}(t)\le N_{GPD, 2}(t)\quad \hbox {for all}\ t\ge 0. \end{aligned}$$

### First-passage-time problem

In Sect. 3.4, we considered the threshold crossing problem for the deterministic growth curve. Now, we analyze the similar first-passage-time problem of the considered stochastic process through certain thresholds. This is important to analyze relevant information on the reaching of critical levels in applied contexts. Let $y \in {\mathcal {S}}$ be the initial value and $k\in {\mathbb {N}}$ a threshold, with $k>y$. Aiming to analyze the first-passage-time random variable

$$\begin{aligned} T_{y,k}=\inf \{t\ge 0:\, X(t)=k\}, \qquad X(0)=y, \end{aligned}$$

we denote by $g_{y,k}(t)=d \,{\mathbb {P}}(T_{y,k}\le t)/dt$ its probability density function (pdf). As in [12], we have

48 $$\begin{aligned} g_{y,k}(t)=(k-1)\lambda (t) P_{y,k-1}(t), \qquad t\ge 0, \end{aligned}$$

where $\lambda (t)$ and $P_{y,k}(t)$ are given in (42) and (39), respectively. For the behavior of $g_{y,k}(t)$ in proximity of $t=0$ one has:

$$\begin{aligned} \lim _{t \rightarrow 0} g_{y,k}(t) =\left\{ \begin{array}{ll} A \,y\,, &{} \quad \hbox {if}\ k=y+1\\ 0,&{} \quad \hbox {otherwise}. \end{array} \right. \end{aligned}$$

**Fig. 20:**
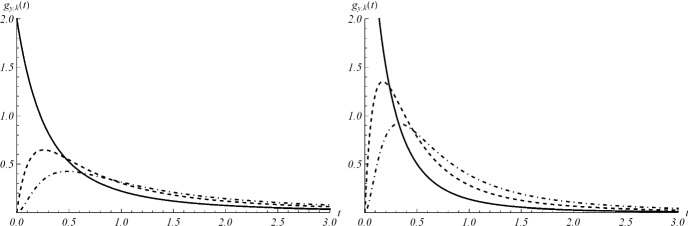
Density (48) for $A=2,\, a=b=2$; on the left: $k=2, \, 3, \, 4$ (solid, dashed, dot-dashed line) and $y=1$; on the right: $k=3,\,4,\,5$ (solid, dashed, dot-dashed line) and $y=2$

**Fig. 21:**
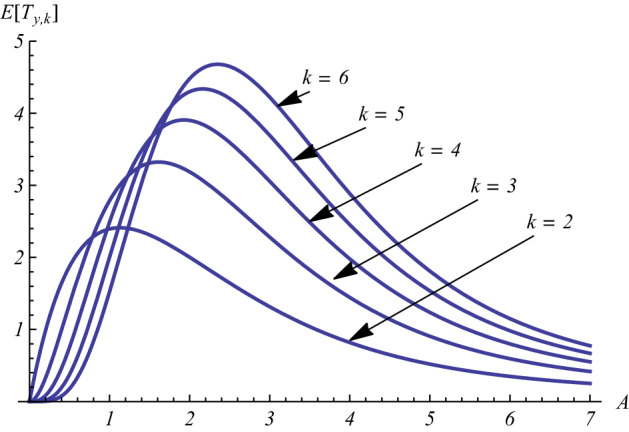
For $y=1$, the mean of $T_{y,k}$ is plotted as function of *A* with $a=b=1$

As example, in Fig. 20, the density (48) is plotted for some choices of the parameters. Moreover, Fig. 21 provides some instances of the mean of $T_{y,k}$.

For the first-passage-time problem of *X*(*t*) through a time-varying boundary we consider the continuous function $t\mapsto \beta (t)$, where $\beta (0)> y$, and define

$$\begin{aligned} T_{y,\beta }=\inf \{t\ge 0:\, X(t)=\beta \}, \qquad X(0)=y, \end{aligned}$$

If $\beta (t)$ is monotone nonincreasing, then similarly as in Proposition 3 of Di Crescenzo and Pellerey [14] we have

$$\begin{aligned} {\mathbb {P}}[T_{y,\beta }>t] =\sum _{n=0}^{\lfloor \beta (t)^-\rfloor } P_{y,n}(t), \qquad t\ge 0, \end{aligned}$$

with $P_{y,n}(t)$ given in (39). In more general cases the determination of the first-passage-time pdf $g_{y,\beta }(t):=d \,{\mathbb {P}}(T_{y,\beta }\le t)/dt$ can be handled by means of computational methods. In the next section we discuss a simulation procedure for *X*(*t*) which can be used to obtain estimates of first-passage-time pdf’s.

### Simulation

For the birth process $\left\{ X(t);\,t \ge 0\right\}$, characterized by time-dependent birth rates (38) and initial state $X(0)=y\in {\mathbb {N}}$, we can construct a customary event-based simulation procedure. Denoting by $T_k$ the *k*-th increment (birth) of the process, with $T_{0}=0$, for all $k\in {\mathbb {N}}$ one has

$$\begin{aligned} {\mathbb {P}} (T_{k+1}>t \,|\, T_{k}=\tau )=e^{-(k+y)[\varLambda (t) -\varLambda (\tau )]}, \qquad t>\tau \ge 0, \end{aligned}$$

where $\varLambda (t)$ is given in (43). This is not a *bona fide* distribution since, due to Eq. (44),

$$\begin{aligned} {\mathbb {P}} (T_{k+1}>t \,|\, T_{k}=\tau )\quad {\mathop {\rightarrow }\limits ^{t\rightarrow +\infty }}\quad e^{-(k+y)[Ab-\varLambda (\tau )]}=: \ell . \end{aligned}$$

Hence, given that $T_k=\tau \ge 0$, the next birth occurs at a finite time $t=T_{k+1}$ with probability $1-\ell$, and thus the simulation of $T_{k+1}$ can be performed through the steps indicated hereafter:

1.: generate a random variable, say *U*, uniformly distributed in (0, 1);
2.: if $U\le \ell$ then $T_{k+1}$ does not occur at a finite time,

else, with *U* taking value in $(\ell ,1)$, set

$$\begin{aligned} t=T_{k+1}=\varLambda ^{-1}\left( \varLambda (\tau )-\frac{1}{k+y}\log U\right) , \end{aligned}$$

where, with $A,a,b>0$, the inverse of $\varLambda (t)$ is given by

$$\begin{aligned} \varLambda ^{-1}(s)=\frac{b}{a}\left[ \left( 1-\frac{s}{Ab}\right) ^{-a}-1\right] , \qquad s\ge 0. \end{aligned}$$

**Fig. 22:**
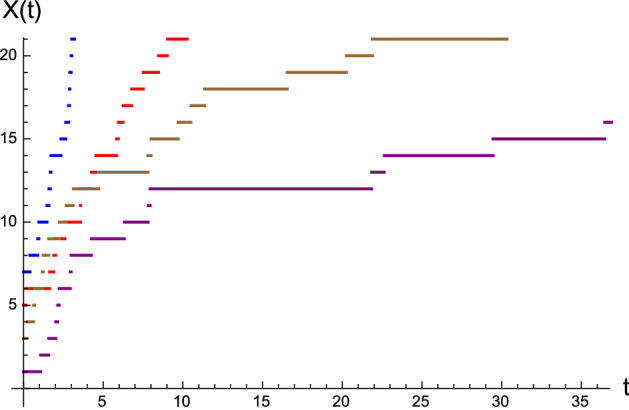
Simulated sample paths of the birth process *X*(*t*), with $A=1$, $b=3$, $(a,y)=(0.5,7)$, (1, 5), (2, 3), (3, 1), from left to right on the top of the figure

**Fig. 23:**
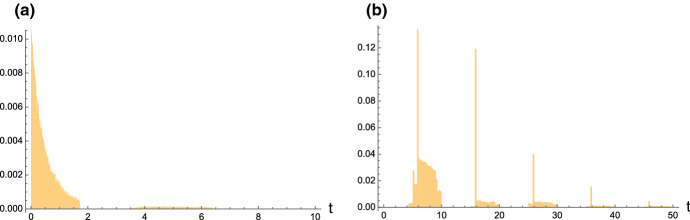
Histograms of simulated first-passage times of the birth process *X*(*t*) through **a** $\beta (t)= \log (t+1)+2$, and **b** $\beta (t)= 2 \sin (\pi t/5)+7$, for $A = 1$, $a = 0.5$, $b = 3$, and $y = 1$

Figure 22 shows examples of simulated sample paths of *X*(*t*) obtained by means of the above sketched procedure. The latter may be used to develop a simulation-based approach to the first-passage-time problem of the birth process *X*(*t*) through a time-varying boundary $\beta (t)$. Indeed, estimates of the pdf $g_{y,\beta }(t)$ can be constructed in terms of histograms obtained by means of extensive simulations based on the above procedure. As example, we consider the same case study treated in Sect. 4.1 of [14], for the boundaries (a) $\beta (t)= \log (t+1)+2$ and (b) $\beta (t)= 2 \sin (\pi t/5)+7$. Estimates of the corresponding first-passage-time pdf’s have been obtained through $10^5$ simulated sample paths of *X*(*t*) performed by use of MATHEMATICA$^{\textregistered }$. See Fig. 23 for the corresponding histograms. As in similar cases exploited in the past, in case (a) the histogram exhibit changes of shapes when the boundary takes integer values, whereas in the case (b) the histogram reflects the periodicity of the periodic boundary. Moreover, we point out that the ratio of simulated sample paths of *X*(*t*) that reach the boundaries in the two cases is (a) 0.033 and (b) 0.202, respectively.

## Concluding remarks

The choice of a suitable curve to describe a growth phenomenon is always a crucial task, since not all features of the relevant physical model and of the observed data can be captured by a given function. In many cases modelers are forced to perform the analysis on the ground of several curves aiming to compare the pertaining results and thus to detect the best choice on the basis of suitable statistical indexes. In some cases the presence of several parameters in the model allow to obtain a better fit of data, but an excess of parameters leads to a lack of correspondence between the considered model and the physical reality of the growth event. Hence, a proper compromise is required between the complexity of the model and its correspondence with the observed phenomenon.

On the ground of these remarks, in this paper we proposed a growth model that provides a suitable generalization of the celebrated Gompertz model, but it is not excessively complex. Indeed, it involves three parameters, other than the initial (positive) value of the growth curve. After a thorough analysis of useful characteristics, we also focused on applications of the growth curve to real data concerning epidemiological and reliability contexts, where the proposed model is found very suitable to describe the observed dynamics under the ISRP metric and the $d_2$-distance. We developed and analyzed two stochastic counterparts of the proposed model. They are based on an inhomogeneous linear birth-death process and a linear birth process. In both cases the correspondence between the growth curve and the mean of such stochastic processes is insured by a special choice of time-varying coefficients for the birth and death rates.
